# Harmonic Training and the Formation of Pitch Representation in a Neural Network Model of the Auditory Brain

**DOI:** 10.3389/fncom.2016.00024

**Published:** 2016-03-23

**Authors:** Nasir Ahmad, Irina Higgins, Kerry M. M. Walker, Simon M. Stringer

**Affiliations:** ^1^Department of Experimental Psychology, Oxford Centre for Theoretical Neuroscience and Artificial Intelligence, University of OxfordOxford, UK; ^2^Department of Physiology, Anatomy and Genetics, University of OxfordOxford, UK

**Keywords:** competitive neural network, auditory brain, pitch identification, harmonic training, unsupervised learning

## Abstract

Attempting to explain the perceptual qualities of pitch has proven to be, and remains, a difficult problem. The wide range of sounds which elicit pitch and a lack of agreement across neurophysiological studies on how pitch is encoded by the brain have made this attempt more difficult. In describing the potential neural mechanisms by which pitch may be processed, a number of neural networks have been proposed and implemented. However, no unsupervised neural networks with biologically accurate cochlear inputs have yet been demonstrated. This paper proposes a simple system in which pitch representing neurons are produced in a biologically plausible setting. Purely unsupervised regimes of neural network learning are implemented and these prove to be sufficient in identifying the pitch of sounds with a variety of spectral profiles, including sounds with missing fundamental frequencies and iterated rippled noises.

## 1. Introduction

### 1.1. Pitch and the auditory brain

**Figure 1:**
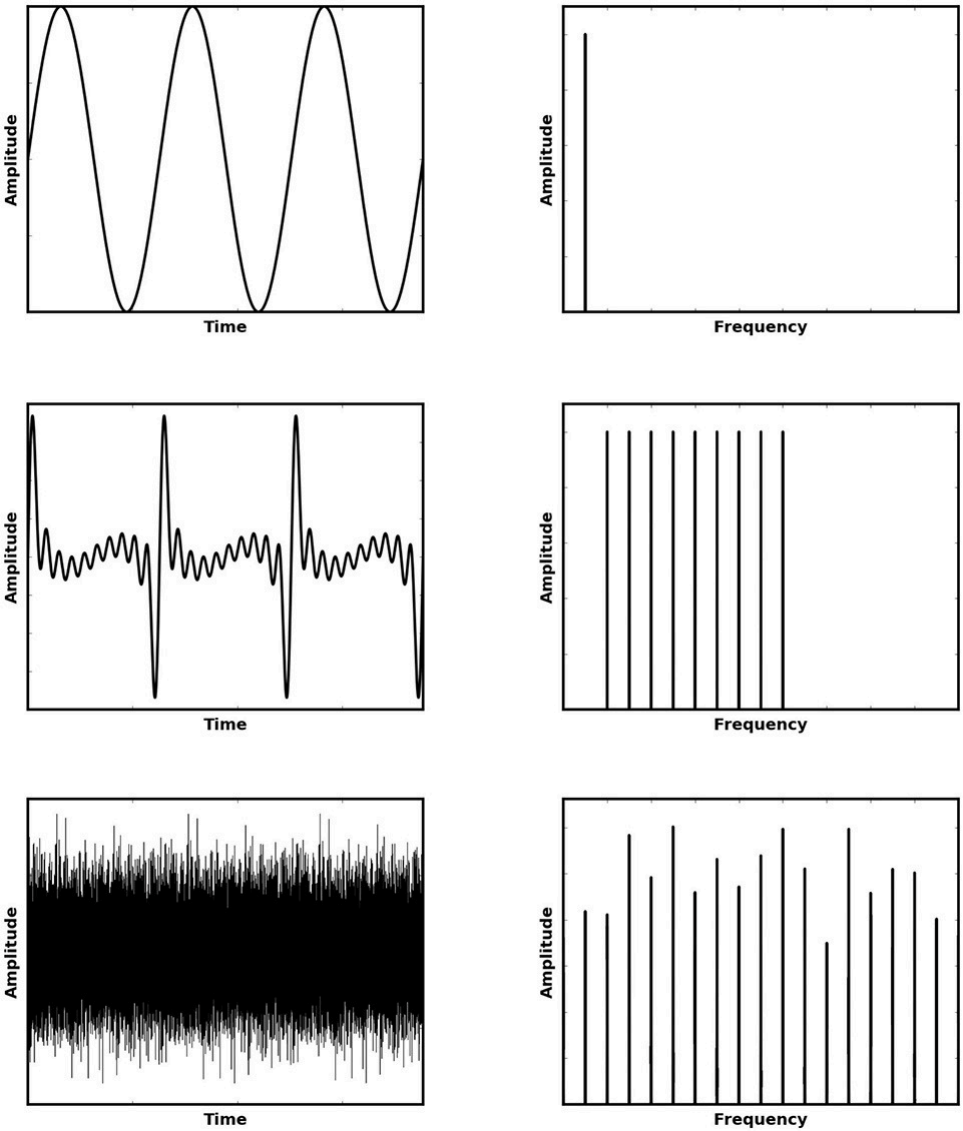
**Various stimuli (left) which elicit a sensation of approximately the same pitch and their corresponding Fourier Transforms (shown right)**. The top row shows a pure tone, the middle row shows the first 10 harmonics of the preceding tone without the fundamental frequency (i.e., a missing fundamental stimulus) and the bottom row shows an Iterated Rippled Noise stimulus (30 iterations of addition).

Pitch, an entirely perceptual phenomenon, conveys a great deal of semantic information in speech, music and animal vocalizations, and plays a key role in our ability to attend to a single object in our noisy natural environments. It can be described as the tonal (low or high) quality of sound, which is most often acoustically associated with the periodicity of that sound. A pitch experienced is generally characterized by its fundamental frequency (F0), the pure tone (Figure 1, top row) which produces the most similar experience. Natural sounds which exhibit pitch can be shown to contain not only a fundamental frequency but also energy in frequency components which are integer multiples of this F0 (i.e., higher harmonics). We shall refer to such pitch stimuli as “F0-containing stimuli.” However, the features which make pitch perception an interesting phenomenon are the full range of instances in which a pitch sensation is elicited despite the sound having very different acoustical characteristics to this well defined case.

An interesting feature emerges when the fundamental frequency of a pitch is removed (Figure 1, middle row). This type of pitch is referred to as a missing fundamental (MF) pitch. Although the resulting sound has energy only at higher harmonics, it nonetheless elicits a perception of pitch at the fundamental frequency, which corresponds to the highest common multiple of the present harmonics. Another interesting sound which is able to elicit a pitch percept is an iterated rippled noise (Figure 1, bottom row) (Yost, 1996). Iterated Rippled Noises (IRNs) are sounds in which broadband noise is added to itself multiple times at a given time delay. This time delay is the period of the resulting pitch experienced by the listener. Thus, as the sound is made up of broadband noise, amplitude variations in the sound waveform remain largely irregular, but become more periodic as the number of delay-and-add iterations are increased. Nonetheless, there is a very clear set of regular peaks in the frequency spectrum and listening to such a sound produces a sensation of pitch.

Sound waves are first transduced into neural signals in the cochlea. Here, the primary auditory nerve fibers act as a gammatone filter bank, each tuned to a different frequency in the sound. This produces a logarithmically-spaced frequency representation of the sound, known as the tonotopic map. Due to the logarithmic spacing, these cochlear filters are broader for higher frequencies, such that only the lower harmonics (approximately the first 6–10 harmonics) of a pitch-evoking sound are resolved on this map (Glasberg and Moore, 1990). Harmonics which lie above this range are generally difficult to distinguish due to the overlap and proximity of cochlear hair cell frequency receptive fields. In order to extract the pitch of the sound, the higher auditory system must integrate information across the cochlear frequency filters in order to compute the spacing between harmonics.

The place theory of pitch suggests that because hair cells and, in turn, auditory nerve fibers, are topographically mapped according to their preferred frequency, pitch is represented as the spatial pattern of excitation across this map (Goldstein, 1973; Cohen et al., 1995). Temporal theories instead point out that as pitch-evoking sounds are periodic, their pitch can be derived from the temporal dynamics of cochlear activation. Due to the ability of neurons in the cochlea and auditory brain to produce action potentials that are phase locked to the envelope of a sound stimulus, the pitch of a sound can be derived as the dominant periodicity of spiking in responses that are pooled across the auditory nerve fibers (Cariani and Delgutte, 1996). The temporal theory can explain how we experience pitch even when only high numbered, unresolved harmonics are present in a sound (Moore et al., 1985; Shackleton and Carlyon, 1994; Bernstein and Oxenham, 2003). However, temporal theory has more recently been argued to be insufficient to provide a complete description of pitch (Shamma, 2004; Oxenham et al., 2011). A combined model of both place and temporal pitch encoding is likely to be necessary to explain the full range of stimuli that evoke a perception of pitch, and these more complex models are being developed (Shamma and Klein, 2000; Oxenham, 2008).

This paper describes how neurons might be expected to form a representation of pitch when provided with input from a biologically realistic cochlear model. The model implemented was based upon information in the place code alone and was not tested to determine how temporal information might contribute to the resulting pitch representation. We propose that a simple system based upon place theory alone can produce neurons that identify the pitch of a range of complex sounds, including missing fundamental and IRN stimuli.

## 2. Methods

### 2.1. Cochlear model

The cochlear model implemented in this paper has been shown to reproduce a number of phenomenon in the auditory nerve. Some of the features which allow it to do so are: two modes of basilar membrane excitation contributing to Inner Hair Cell (IHC) firing [via parallel filters and transduction through separate filters (Zilany and Bruce, 2006)] and power-law adaptation of IHC firing [as shown to be better suited to reproduction of physiological data when compared to alternatives (e.g., exponential adaptation) (Zilany et al., 2009)]. More recently still, tuning parameters based either on the cat cochlea or the human cochlea (Zilany et al., 2014) have been made available. The implementation of the Zilany model was achieved by use of the online available *Cochlea* library (Rudnicki and Hemmert, 2014). In order to produce the response of the cochlea to various auditory stimuli we implemented this as the first stage of the network. For our rate coded implementation, we removed the fine structure of the neural firing patterns and therefore defined the auditory nerve fiber (ANF) firing rates as the average of their temporally varying firing rates over the entire time period of the stimulus.

Our simulations implemented the *Cochlea* model with 2500 ANFs in the range 125 to 20,000 Hz. These ANFs were each normalized and their outputs were provided to the neural network as the set of input neurons.

### 2.2. Network model

**Figure 2:**
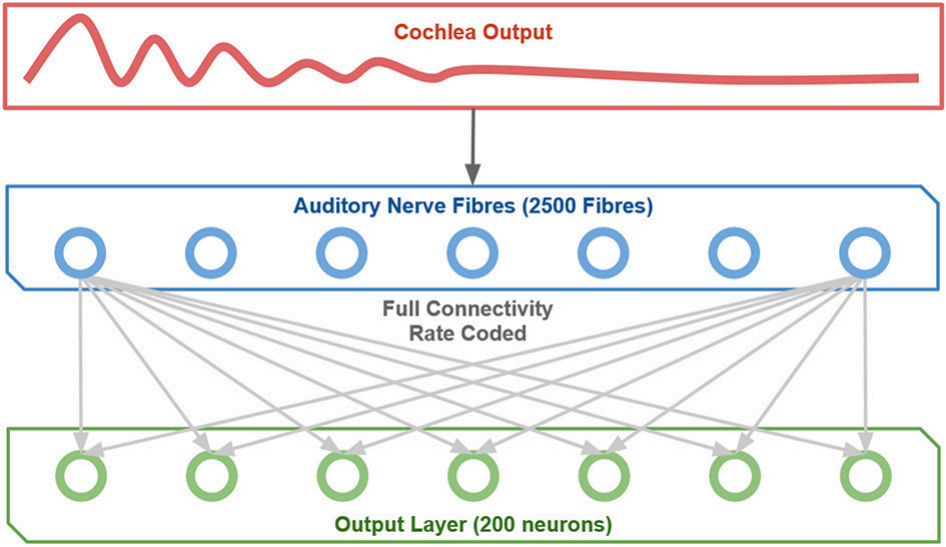
**The network structure implemented**.

The rate coded neural network is a 1-layer model (i.e., one layer of modifiable synapses) with full feedforward connectivity as shown in Figure 2. There is an input layer of cells which represent the output of the cochlea with associatively modifiable synaptic connections onto an output layer of cells. There is competition between neurons in the output layer, which in the brain is implemented by inhibitory interneurons. This mechanism allows the network to self-organize pitch representations in the output layer through associative learning on the afferent connections to these neurons. Thus, the model reflects the general architecture and operational principles of cortex. Furthermore, it has the minimal architecture that captures the essential learning mechanisms demonstrated in this paper that lead to the emergence of representations of pitch.

Individual output cells in this network learn to represent the pitch of the sound stimulus presented to the network through a form of coarse coding. That is, as pitch perception occurs over a continuum, neurons are expected to learn to respond over some interval of pitch with a peaked response curve.

At each time-step during learning, an input pattern is applied to the layer of input cells (the Auditory Nerve Fiber layer). This consists of setting the firing rates of the input cells equivalent to the ANF firing rates produced by the cochlear model. Next, activity from the input layer is propagated through the feedforward connections to activate the cells in the output layer.

The activation of the cells in the output layer are calculated according to

(1) $$\begin{array}{l} h_{i}=\underset{j}{\sum }w_{ij}r_{j} \end{array}$$

where *h*_*i*_ is the activation of output neuron *i*, *w*_*ij*_ is the weight of the synapse from input neuron *j* and output neuron *i*, and *r*_*j*_ is the firing rate of input neuron *j*.

The activation *h*_*i*_ of each output neuron is then converted to their firing rate *r*_*i*_ using a threshold non-linear activation function. This sigmoid activation function has the form

(2) $$\begin{array}{l} r_{i}=1∕\left(1+e^{-2\beta \left(h_{i}-\alpha \right)}\right) \end{array}$$

where *r*_*i*_ is the firing rate of the output neuron *i*, β is the slope of the sigmoid function, α is the threshold of activation of the sigmoid function, and *h*_*i*_ is the activation of the output neuron *i*.

Within the output layer there is competition implemented by shifting the threshold α of the non-linear sigmoid activation function. Adjustment of the threshold is carried out in order to achieve a prescribed sparseness of the representation in the output cells. Sparseness is defined as the proportion [0, 1] of neurons that are active in the output layer. This shifting threshold and defined sparseness represents a process by which mutual inhibition between the output cells through inhibitory interneurons implements competition to ensure that there is only a small winning set of output cells left active. The sparseness was fixed for all simulations at 10%. This was achieved by determining the threshold of the activation function at which this sparseness would be achieved. In this case, as there were 200 output neurons and we desired 10% sparseness, the threshold α of the activation function (1) was set equal to the activation of the output neuron with the 20th highest computed activation *h*. The threshold implemented was the same for all output neurons during a single presentation and redefined on each subsequent presentation of a stimulus. Varying the sparseness had no significant effect upon the network performance and solely altered the number of neurons which collectively responded to a given training stimulus. The learning rate and sigmoidal slope shown in Table 1 were chosen through a grid search of parameter space to find the parameters which resulted in the greatest network performance.

**Table 1:** **The parameters used for the simulations described in this section (except where stated otherwise in text)**.

**Parameter**	**Value**
Learning rate	0.25
Sigmoidal slope	17.45
Sparseness	10%
Number of epochs	50

Next, the synaptic weights between the active input cells and the active output cells are strengthened by associative (Hebbian) learning. The output cells self-organize to represent and thus categorize different patterns of activity in the input layer. The associative Hebb learning rule is defined

(3) $$\begin{array}{l} \delta w_{ij}=kr_{i}r_{j} \end{array}$$

where δ*w*_*ij*_ is the change in the weight between input neuron *j* and output neuron *i*, *k* is the learning rate constant, *r*_*i*_ is the firing rate of the output neuron *i* and *r*_*j*_ is the firing rate of the input neuron *j*. To prevent the same few neurons from always winning the competition, the synaptic weight vectors are set to unit length after each learning update for each training pattern. To implement weight vector normalization the synaptic weights were rescaled to ensure that for each output cell *i* we have

(4) $$\begin{array}{l} \sqrt{\underset{j}{\sum }{\left(w_{ij}\right)}^{2}}=1 \end{array}$$

where the sum is over all input cells *j*. Such a renormalization process was observed in coronal slices of the amygdala by Royer and Paré (2003). Such a mechanism would require interaction between the cells in the output layer and is therefore an augmentation to our proposed connectivity.

The competitive network contained 2500 input cells, 200 output cells and was fully connected. The synaptic weights from the input to output cells were initially set to random values from a uniform distribution in the range 0–1, and then the weight vector of the individual output cells are normalized to a vector length of 1. Each simulation employed a set of stimuli spanning a range of fundamentals from 200 to 600 Hz. These pitch stimuli were individually presented to the network after being processed through the cochlear model. For each individual stimulus, presentation involved calculating the average firing rates of the 2500 Auditory Nerve Fibers (ANFs) in the cochlear model, normalizing across these ANFs, and setting the firing rate of the input cells to the model according to the normalized average firing rate of their corresponding ANF.

Unless stated otherwise, all models described in this paper used the set of parameters described in Table 1.

The presentation of all possible stimulus training patterns (in a randomized order) corresponds to one training epoch. Unless stated otherwise, for each experiment there were 50 training epochs to ensure convergence of the synaptic weights.

### 2.3. Single cell information analysis

A single cell information measure was implemented to analyse output neuron performance (Rolls and Milward, 2000). This measure determined how much information an individual neuron's activation provided about the pitch category to which a given stimulus belonged. The calculation of information is based upon Shannon's information theory, describing the amount of information, *I*(*s, r*), that a set of possible responses, *R*, give about a stimulus, *s*, such that

(5) $$\begin{array}{l} I\left(s,R\right)=\underset{r\in R}{\sum }P\left(r|s\right)\log_{2}\frac{P\left(r|s\right)}{P\left(r\right)} \end{array}$$

where *r* is a response from the set of possible responses, *R*, of a particular neuron. The information content of a single neuron is greatest when it is responsive to all sounds which elicit a given sensation of pitch (associated with some F0) without being responsive to sounds which elicit a different sense of pitch (of a different associated F0). The maximum amount of information, *I*_*max*_, which a single cell can contain is calculated

(6) $$\begin{array}{l} I_{max}=\log_{2}N \end{array}$$

where *N* is the total number of stimuli. Cells will be expected to increase their amount of information significantly through training though they may not often reach this maximum due to the coarse coding discussed above.

### 2.4. Sound stimuli

The sound stimuli presented to the network were of three main categories: F0-containing harmonic tone complexes, missing fundamental harmonic tone complexes and Iterated Rippled Noises. All of these stimuli were produce such that the fundamental frequencies, F0, varied from 200 to 600 Hz in 20 Hz increments. The F0-containing stimuli all contained 10 harmonics (including F0). Missing fundamental stimuli contained no F0 component but the 9 subsequent harmonics. Finally, Iterated Rippled Noises were created by producing a random broadband noise and adding this noise to itself for 30 repetitions (at delays corresponding to the period of the fundamental frequencies 200 to 600 hz). These sounds were all presented to the cochlear model scaled to 50 dB SPL with cosine onset and offset ramps.

**Figure 3:**
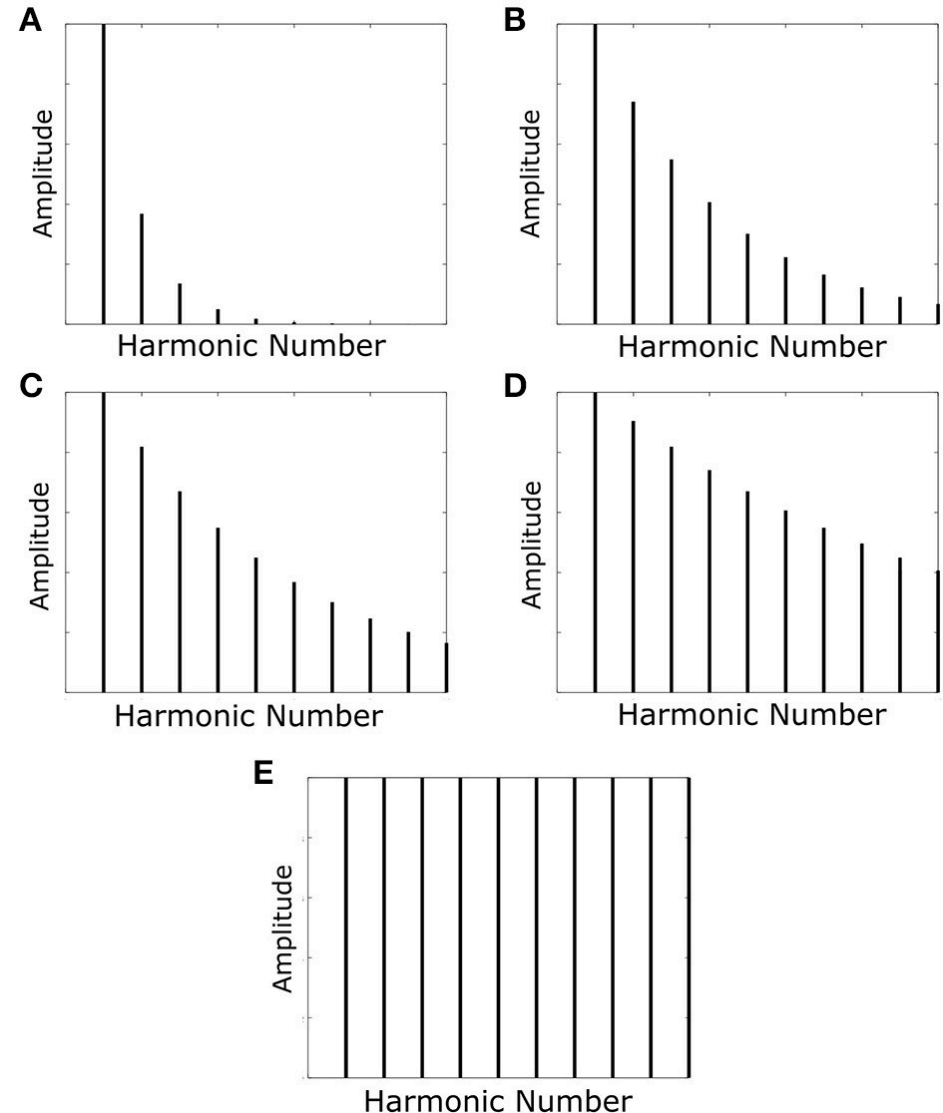
**The harmonic profiles with which the network was trained and tested**. The decays in amplitude of the harmonics are such that their amplitude is multiplied by the function *exp*(−*f*/τ) with varying values of the decay constant τ. Decay constants for the various profiles are as follows: **(A)** τ=*F*0; **(B)** τ=(10/3)*F*0; **(C)** τ=(10/2)*F*0; **(D)** τ=(10)*F*0. Profile **(E)** has no harmonic decay and therefore has a constant amplitude.

The harmonic decay profiles of the harmonics of the F0-containing stimuli and missing fundamental stimuli were the only properties varied. Rather than assuming a fixed harmonic decay profile, we tested the network with a set of variable profiles (Figure 3). These harmonic decay profiles were chosen to test how well the network could recall the pitch of a sound when trained on sounds with varied relative power in their harmonics. During each training epoch, the network was presented with a single set of 21 F0-containing stimuli (all with the same harmonic decay profile) in a randomized order (no pitch presented twice). This presentation was carried out for 50 epochs of training.

The network was finally tested with the same pitches (F0 values) it was trained with. In this case we first tested to see whether the neurons were able to distinguish the different pitches by presenting F0-containing stimuli and observing the output. Finally, missing fundamental stimuli were presented and the output once again observed to check whether our network was capable of correct pitch categorization.

It is important to note that in all results presented hereafter, training was carried out only with F0-containing stimuli.

## 3. Results and discussion

**Figure 4:**
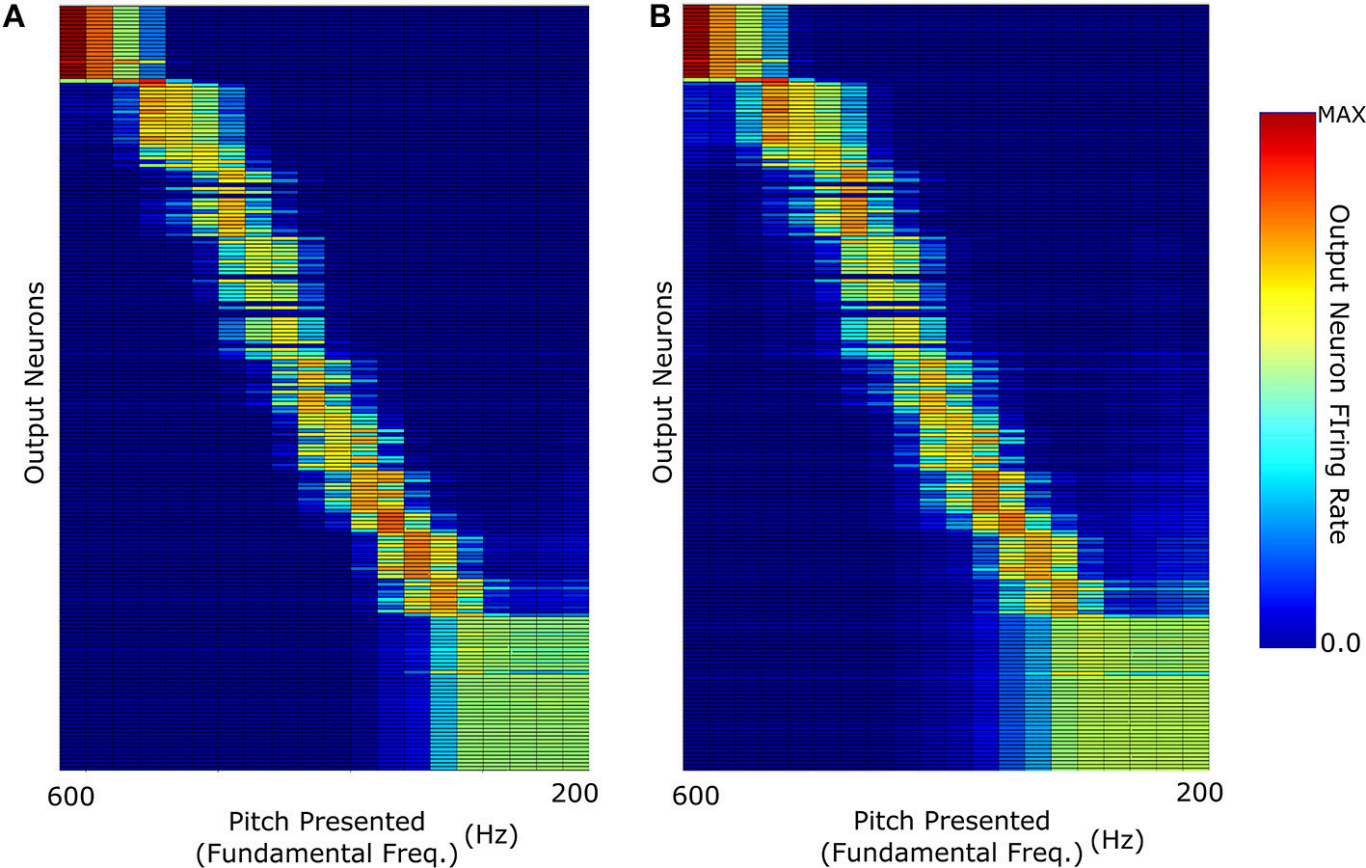
**The neuronal firing rate profiles were sorted along the y-axis after the simulation according to the location of their highest peak**. **(A)** The network response when it was trained and then tested with F0-containing stimuli with no decay in the harmonic profile. **(B)** Response when trained with F0-containing stimuli and then tested with missing fundamental stimuli (again of no harmonic decay).

The key question in this study was whether the network trained on harmonic complex tones that included F0 could generalize pitch categorization to missing fundamental stimuli. We began by analysing the network response when it was trained and tested with no harmonic decay (Figure 3E). The neurons in Figure 4 were sorted according to the locations of their highest peaks in firing rate responses and the “block”-like structure observed in these responses (groups of neurons responding to a given stimulus as a set) is an effect the sparseness value implemented in the network. Figure 4A shows that the unsupervised network was able to produce responses that were tuned to the pitch percepts of harmonic tone complexes after this network was trained with those same stimuli.

We observe that the network produces a fairly clean representation of the range of pitch stimuli presented. This figure shows that the network converts the input frequency representation (which has structured peaks based upon the specific pitch stimulus) into a well defined representation of pitch in which individual output neurons are tuned to particular pitches. This same network when tested with missing fundamental stimuli of no harmonic decay (Figure 4B) is able to accurately identify the corresponding pitch. This was facilitated by the fact that no single harmonic in the training of this network had a greater amplitude than any other. Thus, its training did not place a disproportionate amount of importance upon the (now missing) fundamental frequency.

However, one notable feature (of all the results shown in Figure 4) is the lack of distinct structure in neuron firing below 300 Hz fundamental frequency. Due to the logarithmic distribution of frequencies across the cochlea, pitch stimuli of low fundamental frequencies have highly overlapping higher harmonics. In the case of the lowest frequency stimuli tested, the proximity of their highest harmonics becomes so close that they begin to become indistinguishable in this specific training regime.

**Figure 5:**
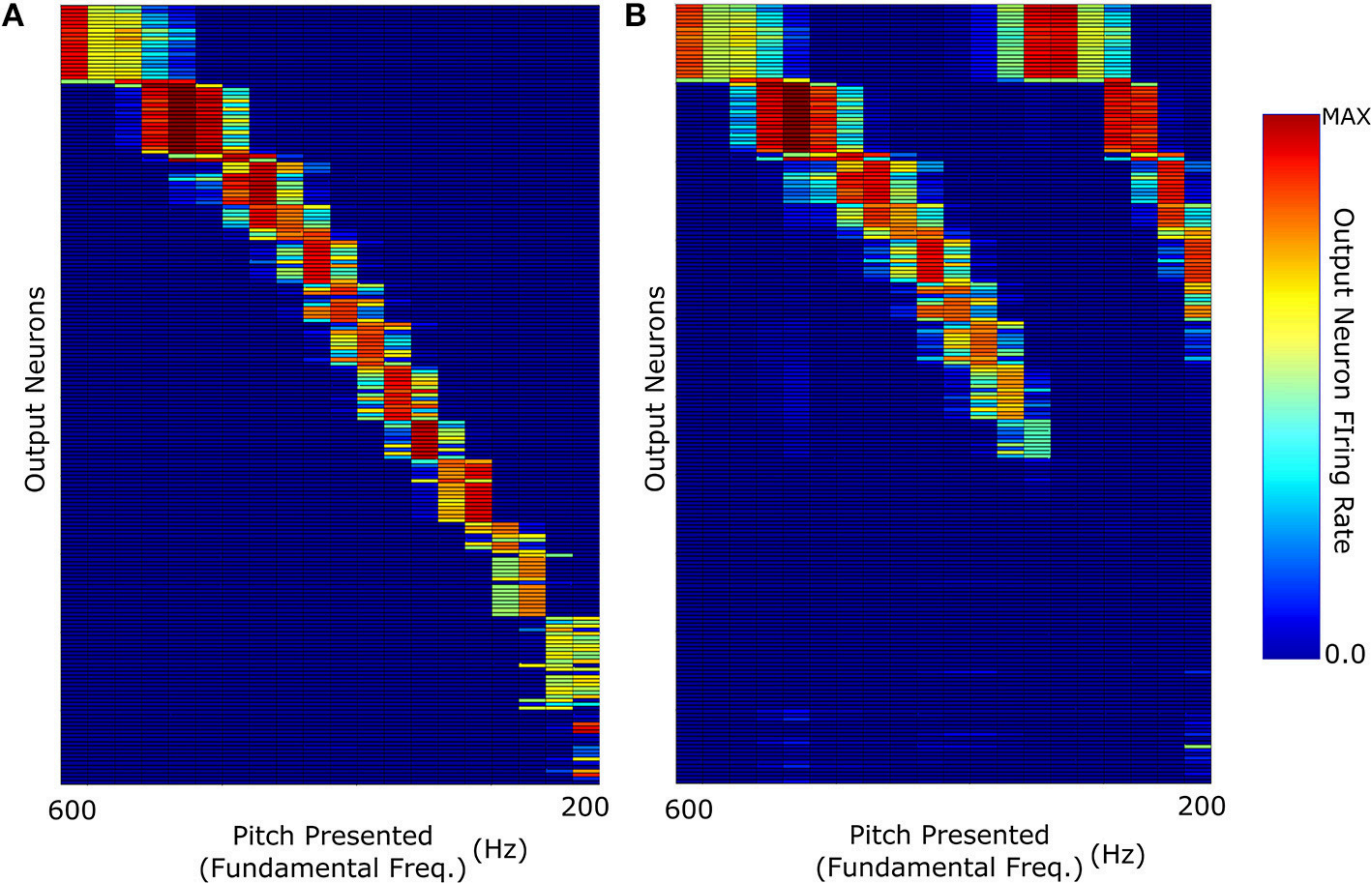
**(A)** The network response when it was trained and then tested with F0-containing pitch stimuli of harmonic profile with exponential decay (τ = *F*0). **(B)** Response when trained with F0-containing stimuli and then tested with missing fundamental pitch stimuli of harmonic amplitude exponential decay (τ = *F*0).

We continued this investigation by training and testing the network with sounds in which the fundamental frequency of the pitch was greatest in amplitude. The other harmonics were damped with amplitudes modified by an exponential decay with decay constant equal to the fundamental frequency of the pitch (Figure 3A). Figure 5A shows the network's ability to identify F0-containing pitch stimuli. The network's ability to distinctly represent stimuli with fundamental frequency greater than 400 Hz appears to be similar to the network performance when trained on stimuli of no harmonic decay (Figure 4). However, at the lower fundamental frequency range (< 300 Hz) this training regime appears to produce a network response much more able to distinguish pitch stimuli. Thus, a network trained and tested on pitches with sharply decaying higher harmonic amplitudes outperforms the network trained and tested on constant harmonic amplitudes (Figure 4A) when tested on F0-containing stimuli of low fundamental frequency (< 300 Hz).

However, as can be observed in Figure 5B, training with decaying harmonic amplitudes does not allow a generalization of pitch representations from harmonic tone complexes to missing fundamental sounds. Training the network with stimuli so heavily weighted to their fundamental frequencies results in a network almost solely dependant upon that frequency. Thus, removal of the fundamental frequency means that the remaining sound is characterized by the next harmonic of highest intensity (i.e., double the fundamental frequency). This results in each pitch stimulus appearing to have a fundamental frequency that is double the true fundamental frequency and the network misidentifies the pitch of the missing fundamental stimulus as a single octave above what it should be. For example, neurons tuned to a pitch of 400 Hz in harmonic tone complexes containing F0, respond to missing fundamental sounds with a pitch of either 400 or 200 Hz. Thus, training a network with stimuli of sharp harmonic amplitude decay does not lead to the development of output neurons that correctly identify missing fundamental stimuli. In this respect, constant amplitude training and testing performs more accurately (Figure 4B).

In testing and training the above described networks, the question arose as to what harmonic decay profile would be the limit beyond which a missing fundamental stimulus would give rise to an incorrect pitch assignment. This was investigated by training and testing the network with stimuli of different harmonic decay profiles as shown in Figures 3B–D. It was found that the harmonic profile for which the decay of the harmonic amplitudes followed *exp*(10*f*/*F*0) (Figure 3D) was the point of change. A larger decay constant allows the neurons to learn a more accurate identification of the MF pitch stimulus. A smaller harmonic decay constant leads to the network misidentify low frequency missing fundamental stimuli (< 400 Hz) as one octave too high.

In order to replicate the mechanisms by which the brain might learn pitch stimuli more realistically, the network architecture was trained with pitches of harmonic decay which were randomly varied across stimuli on every epoch of training for 100 epochs. The variation in the harmonic decay was achieved by setting the decay constant τ = *F*0 · *exp*(*x*) where *x* was a randomly chosen real number between zero and ten for each pitch. Thus, the training stimuli varied from sharp exponential decays to almost constant amplitude stimuli. It is important to note that having the decay constant chosen randomly from an exponential distribution resulted in training with many instances of sounds with a large decay constant and fewer instances of sounds with very short decay constants. This style of training was required in order to develop the results described. In this regime, the stimuli were different for every single epoch with no sound (of a given decay) repeated during training.

**Figure 6:**
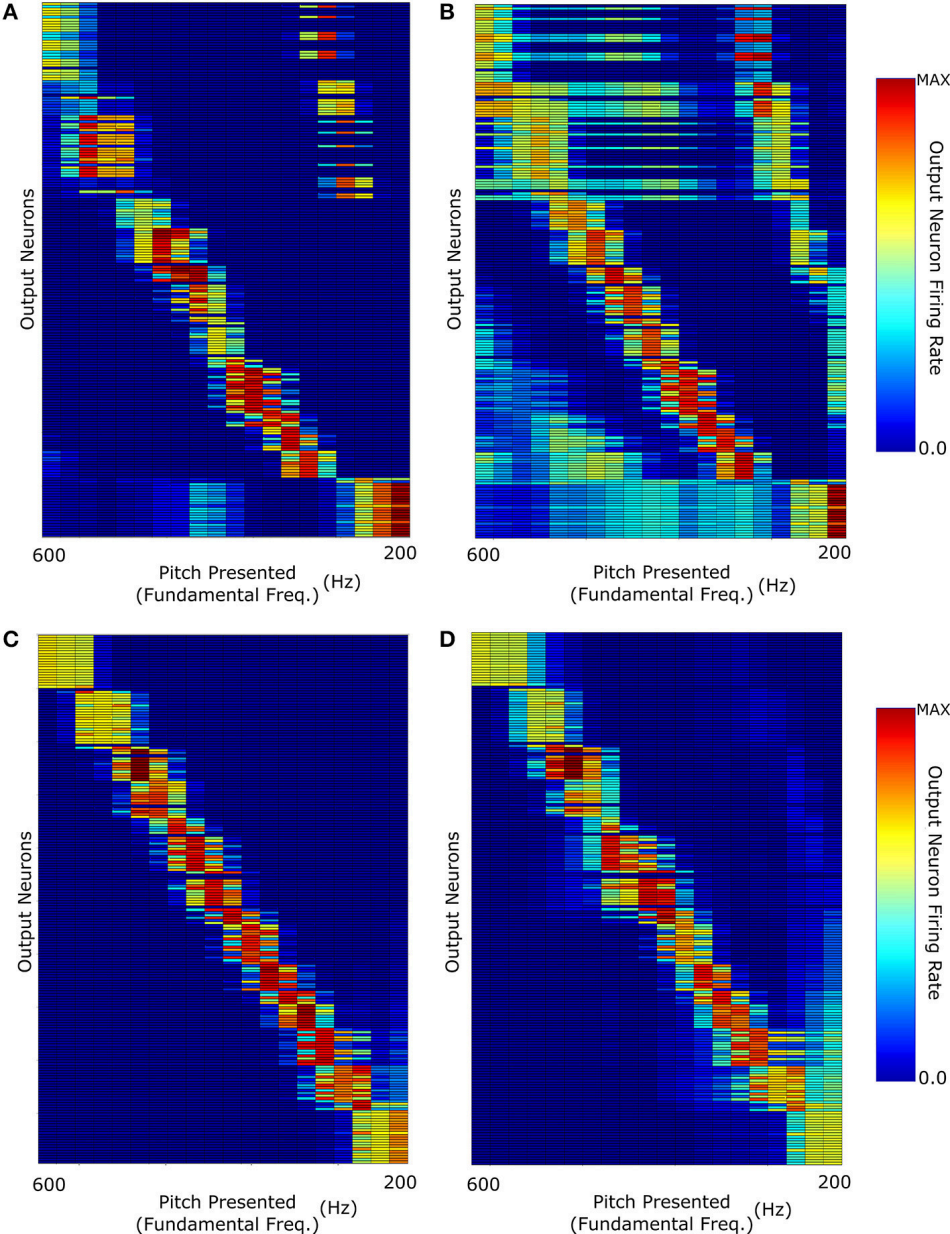
**The network was trained with pitches of randomly selected harmonic decay constants**. The network responses when testing the network with: **(A)** F0-containing stimuli with Harmonic decay constant τ = *F*0; **(B)** The same stimuli as **(A)** but with missing fundamentals; **(C)** Constant Harmonic Amplitude stimuli; **(D)** The same stimuli as **(C)** but with missing fundamentals.

This training resulted in a network response (Figure 6) capable of identifying both decaying and non-decaying harmonics with varying degrees of accuracy for F0 containing and MF stimuli. Crucially, exponentially decaying and constant amplitude stimuli performed close to equally well in representation of F0 containing stimuli. The network shows a greater discrimination of stimuli with low fundamental frequency (< 300 Hz) when tested with constant amplitude pitches that in previous training cases (Figure 6C compared to Figure 4A). It is proposed that this performance increase is due to a more appropriate distribution of synaptic emphasis upon the fundamental and higher harmonics of the stimuli as a result of the training regime. Another important observation is that the exponentially decaying stimuli have a much less structured MF stimulus response, though the issue of pitch octave misidentification has been somewhat addressed. This result emphasizes the potential importance of training of the auditory brain with pitches of differing harmonic decay profiles. The biological relevance of this is something which could potentially be tested in psychoacoustic experiments.

**Figure 7:**
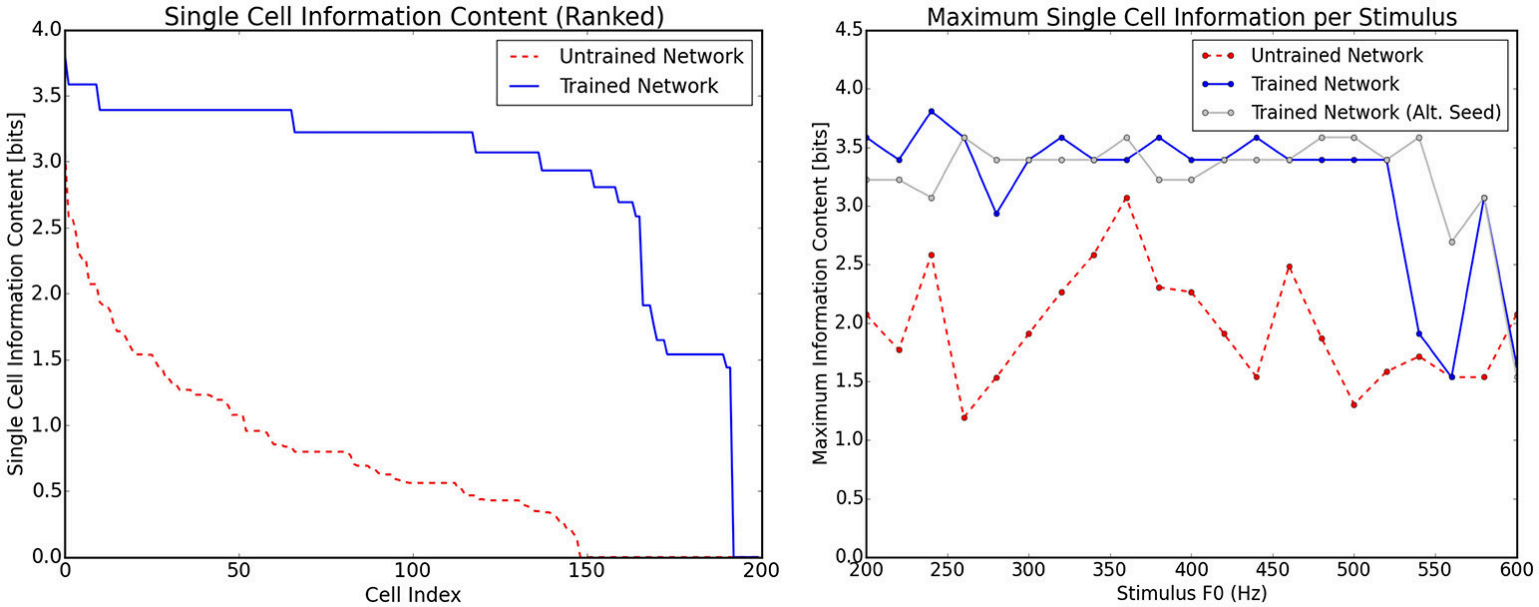
**Information theoretic analyses for the network trained on randomly selected harmonic decay profiles**. The analyses are calculated using the responses shown in Figure 6. Left, the ranked single cell information for the output neurons before and after training. Right, the maximum amount of information that any single cell has with respect to each stimulus in the investigated frequency range. The trained and untrained cases are shown with analysis of a network trained with a different set of initial random weights (with a different seed for the number generator). This illustrates that most of the variation in information is due to the specific random seed.

When single cell information theoretic analyses were applied to the data shown in Figure 6 it was found that there was a significant increase in single cell information from the untrained to the trained case (Figure 7, left) as expected. This analysis was applied by binning the responses of each cell to each stimulus into one of two types. All cells with firing rate greater than 0.5 were placed into one bin and cells with lower than 0.5 firing rate into the other. These bins essentially described (by a very coarse separation) whether a cell was in one of two states: active or inactive. With this assignment of neuron state, the information present in single neurons with respect to stimulus frequency was also shown to be very high for the majority of the frequency range (Figure 7, right). This plot also shows a comparison of the network being presented to an identical network in which the initial random weights were drawn with a different random seed. As can be observed, most of the variation in the information across stimuli can be attributed to the specific initial weight distribution. For frequencies above 540 Hz, there is some drop in single cell information which can be traced to the response of these cells to adjacent pitch stimuli being very similar to one another. Stimuli in this region are however distinguishable by firing rate and therefore this coarse separation of cells into simply firing/not firing is a simplification. In a much larger network (with more coverage of different initial weight combinations), the variations observed in the information per stimulus type would be less apparent and more informative neurons emerging more readily.

**Figure 8:**
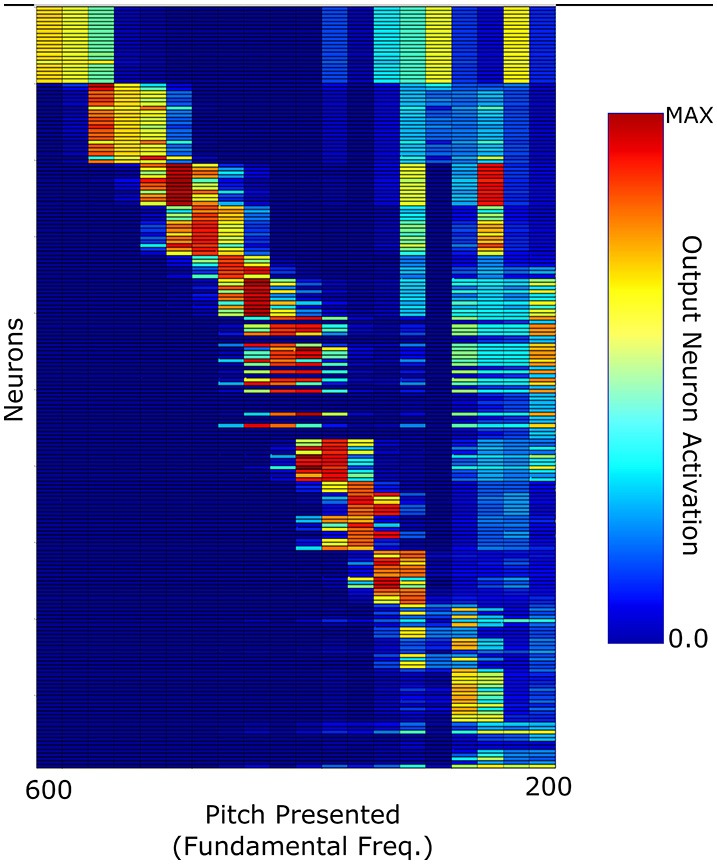
**The network response to Iterated Rippled Noise stimuli when the network was trained with harmonic tone complexes of randomly selected harmonic decay constants**.

**Figure 9:**
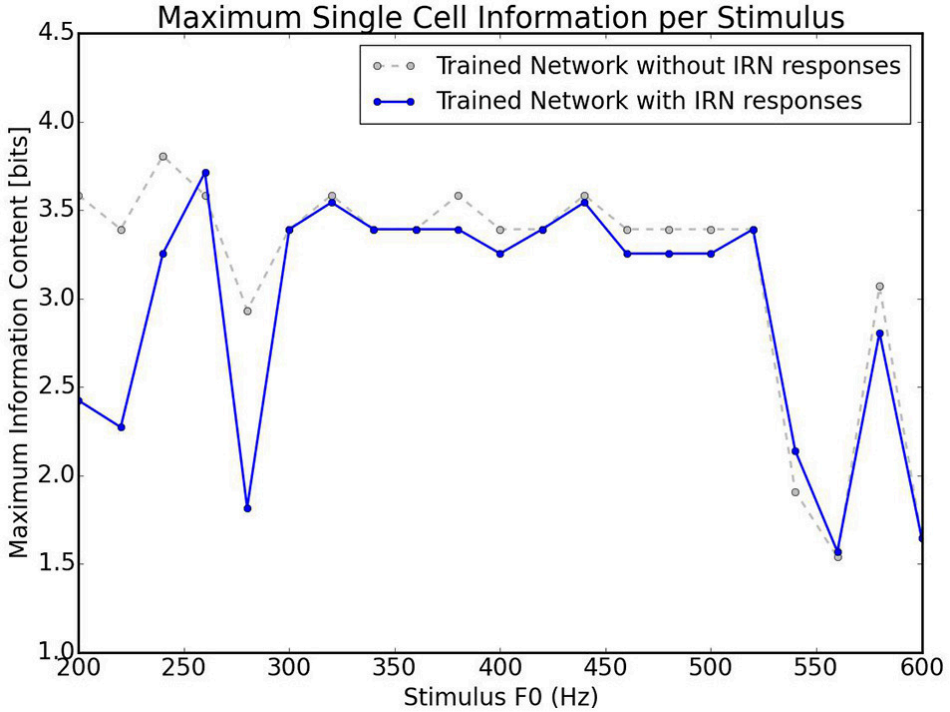
**Information theoretic analyses for the network trained on randomly selected harmonic decay profiles**. Calculated using the responses shown in Figures 6, 8. This plot shows the maximum amount of information that any single cell has with respect to each stimulus in the investigated frequency range. The trained network is analyzed with the responses in Figure 6 and with/without the responses in Figure 8. As can be observed, including IRN responses only reduces the information content of cells informative at low frequencies.

A further test carried out with this network was how well it could categorize the pitches of Iterated Rippled Noises (IRNs) (Figure 8). The network was tested and trained as described above (randomly chosen harmonic decay profiles) and is the same network used to produce Figure 6. Despite the network having never been trained with IRNs, the output shows an ability to separate the IRN stimuli into the various pitch stimuli in a similar arrangement to that seen for the more conventional pitch stimuli. The occasional anomalous behavior may be attributed to the fact that the network was only trained with 10 harmonics for each pitch and the IRN stimuli contain frequency components at many higher harmonics. In order to assess this network's ability to identify individual IRN stimuli, the single cell information theory was re-calculated with the IRN stimuli responses included. As can be observed in Figure 9, including the IRN responses does not significantly affect the network's information content. The lowest fundamental frequency (< 300 Hz) stimuli are observed to have a reduction in information. This is attributed to the the fact that the network was not trained on any more than 10 harmonics (whereas the IRNs had many more than 10 harmonics) and low F0 pitches have high harmonics in very close proximity. Therefore, the network performance when tested upon IRNs is close to equal that of its response to more typical pitch associated sounds (complex tones).

Overall, the network described in this paper shows a reasonably good ability to categorize the pitches of both MF stimuli and IRNs despite never having been trained with examples of these sounds. Furthermore, since this network uses no temporal cues to categorize pitch stimuli, the results support a place theory of pitch (see Introduction) via a very simple mechanism.

Relatively few Neural Network based models have been published in relation to mechanisms of pitch decoding. Among these, fewer still have been based upon unsupervised learning rules. In some cases (Sano and Jenkins, 1989; Taylor and Greenhough, 1994), a supervised network with backpropagation of error was implemented and tested. Such a method of learning requires that specific neurons are “informed" that they must respond to a given stimulus and the neuronal weights are altered to ensure that this is true. This appears too far removed from the neural and synaptic dynamics of the brain to be plausible. Furthermore, the fact that other primates and mammals have perceptions of pitch means that it is difficult to prove that external supervision could drive the development of such a network.

Recently an attempt was made to implement an unsupervised self-organizing map (Zarras et al., 2012). This model was able to form a fairly well defined representation of absolute frequency when trained with pure frequencies. After this training, the network was then tested with F0-containing harmonic tone complexes and missing fundamental stimuli and was not able to distinguish the pitches well and entirely unable to identify MF pitches. The network was then trained with harmonic tone complexes including their F0. The output network learned to classify the pitch of these harmonic tone complex, but only for higher pitched sounds. However, the model was not tested on MF stimuli or IRNs, so it is unclear if the network's pitch rules could generalize to other types of pitch-evoking sounds.

Another recent and particularly interesting attempt to represent pitch in an unsupervised network is an implementation of a spiking neural network (Laudanski et al., 2014). This paper implemented both a place and temporal code in order to produce a representation of pitch. In this case, ANFs from all cochlear locations responding to the harmonics of a pitch were connected to a given co-incidence detector neuron by axons with finite and fixed delays based upon the expected delay between the phases of each harmonic. The fixing of axonal delays was an artificial means of producing these pitch representing co-incidence detector neurons, and it is not clear that such axonal delays exist in the auditory system. Furthermore, while selectively connecting ANFs that are tuned to related harmonics provides a solution for harmonic binding of pitches, there is not yet evidence for this anatomical arrangement in the auditory system.

In this paper we have used a biologically plausible cochlear model as an input (Zilany et al., 2009, 2014) and investigated whether an entirely unsupervised learning network in order to investigate whether a simple arrangement of neurons could form a representation of pitch and recognize missing fundamental sounds or IRNs. We found that training the network on F0-containing harmonic tone complexes with a range of harmonic decay profiles is key to the ability of the network to learn the robust determination of pitches when MF stimuli or IRNs are to be deciphered.

## 4. Conclusion

In this paper, we attempt to create the first entirely unsupervised competitive neural network structure which can learn to identify F0-containing stimuli, Missing Fundamental (MF) stimuli, and Iterated Rippled Noises (IRNs) when presented with biologically realistic inputs from a simulated cochlea. This network is found to perform the task of pitch identification well following training but its performance is found to depend upon the decay profile of the harmonics with which it is trained. In particular, when identifying missing fundamental pitch stimuli, the network requires that the sounds it is trained upon have higher harmonics of amplitude similar to that of the fundamental. Single cell information analysis also shows a high level of information almost the entire stimulus range, including Iterated Rippled Noises. It is concluded that learning pitch in such a network requires exposure during training to stimuli with many different harmonic decay profiles but with a bias toward equal amplitudes of higher and lower harmonics (as implemented with the decay constants chosen from an exponential distribution). Furthermore, the relative success of this network suggests that for pitch stimuli in this frequency range, a place theory of pitch provides a significant amount of information.

## Author note

The code used to create the simulations presented in this paper is available upon request. Details of the model are also fully outlined in the methods section of this paper.

## Author contributions

NA: Carried out the modeling and created figures. IH: Introduced NA to the project, directed and guided through the details of the model. KW: Co-supervisor to NA. Helped in writing/guiding the drafts of the paper, directed in terms of auditory literature and details. SS: Supervisor to NA Explained Network Principles, Idea and guided the writing process.

### Conflict of interest statement

The authors declare that the research was conducted in the absence of any commercial or financial relationships that could be construed as a potential conflict of interest.
